# Prevalence and factors associated with anemia among pregnant women during the COVID-19 pandemic in East Lombok district, Indonesia

**DOI:** 10.1371/journal.pone.0323942

**Published:** 2025-06-25

**Authors:** Amanda Safiera Ameline, Dian Novita Chandra, Min Kyaw Htet, Nur Lailatuz Zahra, Umi Fahmida

**Affiliations:** 1 Department of Nutrition Science, Faculty of Medicine, Universitas Indonesia-Dr. Cipto Mangunkusumo General Hospital, Jakarta, Indonesia; 2 Southeast Asian Ministers of Education Organization Regional Centre for Food and Nutrition (SEAMEO RECFON) - Pusat Kajian Gizi Regional (PKGR) Universitas Indonesia, Jakarta, Indonesia; 3 Sydney School of Public Health, The University of Sydney, New South Wales, Australia; 4 Sinergi Qalbu Fikri, Depok, Indonesia; National Institute of Child Health and Development, JAPAN

## Abstract

**Background:**

Maternal anemia is a major public health problem that has detrimental effects on pregnancy and birth outcomes. The threat of food insecurity and nutritional deficiencies is growing as a result of the COVID-19 pandemic. The aim of this study was to determine the prevalence and risk factors of anemia among pregnant women in East Lombok during the pandemic.

**Materials and methods:**

This community-based cross-sectional study is part of an observational cohort study entitled “UKRI-GCRF Action Against Stunting Hub (AASH)” in rural areas of East Lombok, Indonesia. Data were collected from women (18–40 years) in their third trimester pregnancy (N = 446) from June 2021 to January 2022. Data collection included hemoglobin, mid-upper arm circumference, intestinal helminth infections, and structured questionnaires for sociodemographic, dietary diversity, pregnancy-related, food security (US-HFSSM), reduced coping strategy index (rCSI), and COVID-19-related variables. The association between anemia and its determinants was analyzed using binary logistic regression model.

**Results:**

Anemia was found in 40.8% of pregnant women in third trimester. During the pandemic, 74.7% and 28.9% of women reported a decrease in household income and food expenditure whereas 47% of them reported having medium-to-high coping strategies. The proportion of women who had chronic energy deficiency (CED), inadequate dietary diversity, non-use of contraceptives was 10.5%, 28.3%, and 38.8%, respectively. In logistic regression, anemia was significantly associated with CED (AOR = 1.92; 95%CI: 1.06–3.48), inadequate dietary diversity (AOR = 1.58; 95%CI: 1.02–2.45), and non-use of contraception (AOR = 1.58; 95%CI: 1.02–2.45).

**Conclusions:**

The prevalence of maternal anemia was high in the study area, and it was associated with CED, inadequate dietary diversity, and non-use of contraception. The findings highlight the importance of improving anemia control programs among pregnant women through nutrition education focusing on food based dietary recommendations, and conditional cash transfer which include family planning and compliance of iron folic acid to strengthen their resilience to natural phenomenon such as COVID-19 in rural areas like East Lombok.

## Introduction

Maternal anemia remains a major public health problem in most developing countries [1], including Indonesia [2]. Anemia can have adverse impacts on both maternal and child health, resulting in premature delivery, intrauterine growth restriction (IUGR), low birth weight (LBW), and perinatal mortality [2,3]. The Indonesia Basic Health Survey 2018 was the most recent national data on nutritional anemia. The findings showed that 37.1% of pregnant women were anemic in 2013 [4], which increased to 48.9% in 2018 [5]. In some provinces such as West Nusa Tenggara Province the prevalence was much higher, i.e., 56.5% in 2013 [6]. These figures on anemia prevalence exceed 40% i.e., which WHO considers as a severe public health significance [7].

The COVID-19 pandemic and lockdowns have impacted household income and food security [8], particularly as a result of unexpected increases in unemployment, income shocks, decreased availability of food in local markets, and higher prices for a variety of food groups [9]. Based on the Global Food Security Index (GFSI), Indonesia ranks 63^rd^ out of 113 countries in 2022 and is categorized as a country with moderate food security. The GFS index is basically calculated based on four pillars, including affordability, availability, quality and safety, and sustainability and adaptation. For the dietary diversity indicator (within the quality and safety pillar), Indonesia ranks 109^th^ out of 113 countries (“very weak” score of 34.6) [10].

Inadequate dietary diversity is associated to micronutrient deficiency (i.e., iron, vitamin A, folate, B-12) [11]. Pregnant women in impoverished communities have been reported to consume predominantly plant-based diets that are monotonous and less diversified in vegetables, fruits, and animal sources, contributing to low bioavailable iron and high iron-absorption inhibitors (e.g., phytate and polyphenols) [11,12] which are risk factors of anemia. Women living in poor communities are also at risk of having intestinal parasite infections [13] and poor nutritional status [11], which further increase risk for anemia.

Poor households who experience food insecurity may have worries that food would run out, cut size or skip meals, could not afford balanced meals, or even not eat whole day [14]. Food insecure households are reported to employ a variety of coping strategies, including dietary and financial compromises, as resilience in the early stages of food insecurity when resources are limited or absent [15], and these approaches emerged following a crisis, notably the COVID-19 pandemic. In the short term, this may result in a shift in dietary practices and livelihood patterns [16], yet in long run, it may have a negative impact on health and nutritional status, including maternal anemia. Investigating anemia prevalence and its determinants during the pandemic is therefore critical to identify factors associated with anemia during the shock period such as COVID-19 pandemic to strengthen anemia reduction program.

To the best of our knowledge, there is a limited published literature, especially community-based studies in Indonesia that assess the risk factors of anemia among pregnant women during the COVID-19 pandemic. Therefore, this study aimed to determine the prevalence and risk factors associated with anemia among pregnant women in East Lombok, Indonesia during the COVID-19 pandemic.

## Methods

### Study design and population

This cross-sectional study is part of an observational cohort study in East Lombok, entitled “UKRI−GCRF Action Against Stunting Hub (AASH).” The AASH study aims to investigate the interdisciplinary factors contributing to child stunting using a ‘whole child’ approach. It includes assessments during pregnancy, lactation, and the complementary feeding phase to identify key determinants of child growth and development [17]. For AASH Indonesia, pregnant women in their second trimester were recruited between 12th February and 30th September 2021. In this study, data collection included women in the third trimester of pregnancy, which was conducted from June 2021 to January 2022. East Lombok is a district located in Lombok Island, West Nusa Tenggara Province (also known as *Nusa Tenggara Barat* or NTB), Indonesia. It is the most populated district in NTB, with an estimated population of 1,200,612 in 2019. The administrative area of the district consists of 21 sub-districts and 254 villages. There are 32 public health centers, three hospitals, a few private medical clinics, and over 1,000 active integrated healthcare service posts (referred to as *Posyandu* in Indonesian) [6].

Our study participants were pregnant women in their third trimester, aged between 18 and 40 years old, domiciled in East Lombok and of Sasaknese ethnicity. Pregnant women who were expecting multiple births or had consanguinity were excluded.

### Sampling procedures

The sample size was determined using the single population proportion formula [18], with a 95% Confidence Intervals (CI), 5% margin of error, and anemia prevalence (49.7%) based on a previous community-based study in Indonesia [19]. A minimum sample size of 422 pregnant women was required after assuming a 10% non-response rate and a design effect of 1.0 (since identical or homogeneous samples are expected in the large observational cohort study.

East Lombok district was purposively selected for AASH study due to its high stunting prevalence (above 30%). Among the twenty-one subdistricts, these four subdistricts (Aikmel, Lenek, Sikur, and Sakra) were selected as our study area given the high stunting prevalence (above 30%) [5], representation of different geographic typology, e.g., mountainous and coastal areas, and access to the laboratory within two hours from the time of biological sample collection. In the main study, which will be reported separately, the pregnant women were assigned into intervention arm and to the control arm [20]. This current cross-sectional study only included participants from the control arm.

### Data collection

#### Structured questionnaire.

In this study, structured questionnaires were utilized to collect data on background characteristics (i.e., age, education level, employment status, household wealth index, family size, smoking status); dietary factors (i.e., food security, food taboos and aversions, appetite status, dietary diversity); and pregnancy-related factors (i.e., use of contraceptives, planned pregnancy, parity, number of ANC visits, IFAS consumption adherence), as well as factors relevant to the COVID-19 pandemic (i.e., changes in household income and food expenditure, and coping strategies). Enumerators were recruited from local nutrition academies and were trained for data collection. They were trained for electronic data collection using tablets (commcarehq.org).

The majority of the questionnaires used in the study were standard and widely applied in other studies and national surveys, such as the National Socioeconomic Survey (SUSENAS), Indonesian Basic Health Survey (RISKESDAS), and the cohort study in East Java, Indonesia (BADUTA project) [21]. The researcher (bilingual) performed the translation of questionnaires from English into Indonesian. This version was then piloted for further improvement. Following the pilot study, any doubts or difficulties in responding to the questions were investigated. Another translator did the subsequent back-translation into English. All translators were proficient in English. The final questionnaires were prepared in both Indonesian and English versions. The research team has also adjusted these questionnaires into the local language. The enumerators who collect data should be Lombok natives (able to speak both Indonesian and Sasak) and familiar with the cultural context of the study population, which aided in effective communication and ensured that the question items were delivered accurately. The food insecurity survey tools, such as US-HFSSM and RCSI questionnaire have been validated and used in many countries, including Indonesia [22]. Cronbach’s alpha was calculated to examine the internal consistency of each questionnaire. The US-HFSSM showed strong internal consistency with a Cronbach’s alpha of 0.86, while the RCSI had an acceptable value of 0.7 [23].

#### Food security and coping strategies assessment.

The US Household Food Security Survey Module (US-HFSSM) which consists of 18 question items, was used to determine the household food security status. Respondents are asked about their household food access, availability, and utilization, in the preceding 12 months due to a lack of financial resources to purchase food. The affirmative response (i.e., “yes”; “often”; “sometimes”; “almost every month”; and/or “some months but not every month”) to each question item earning a one-point score. The category of marginal to high food security (score 0–2) is referred to as food secure, while the category of low to very low food security (score ≥3) is referred to as food insecure [24].

The Reduced Coping Strategy Index (RCSI) is a validated instrument, adapted by the World Food Programme, used to assess the coping strategies and behaviors that individuals and households adopt during food shortages experienced in the past seven days [25]. The tool measures food availability, acceptability, quantity, economic accessibility, and even food quality. It aims to identify households facing food insecurity, explore the underlying causes and consequences, and facilitate monitoring and impact evaluation [26]. RCSI includes five behaviors, and respondents were asked how many days (between 0 and 7) in the previous week they engaged in each strategy, which is then multiplied by its severity weight [27]. The overall RCSI score is divided into two main categories: Low coping (0–4) and moderate to high coping strategies (≥5) [25].

#### Wealth index calculation.

The principal component analysis (PCA) based on the USAID dataset of the 2017 Indonesia Demographic and Health Surveys (DHS) Program was used to calculate the wealth index in the current study [28]. The household wealth index is constructed using several variables, which included housing amenities, household assets, and mode of transportation. If the household has or owns the variables, it was classified as “1,” and if not, it was classified as “0.” It was then multiplied by the component score coefficient obtained from the dataset. The results of each variable were added to determine the overall scores. Finally, the household wealth index scores were divided into three equal classes (terciles) for the purposes of classification: poor, middle, and rich [29].

#### Dietary diversity assessment.

Dietary diversity was obtained from 24-h dietary recall with a four-pass multistage interviewing technique [30]. This method was employed since it is less prone to recall bias, and is less burdensome for respondents. Each study participant was asked to list and describe the ingredients of all food and beverages consumed the day and night prior to the survey. The results were then coded and categorized into 10 food groups, including (1) starchy staples (e.g., grains, white roots, tubers, and plantations); (2) pulses (beans and peas); (3) nuts and seeds; (4) milk and dairy products; (5) meat, fish, and poultry; (6) eggs; (7) dark green leafy vegetables; (8) vitamin A-rich fruits and vegetables; (9) other vegetables; (10) other fruits. Participants received a score of 1 if they consumed at least 15 grams of each group; otherwise, they received a score of 0. The dietary diversity score of 10 food groups was calculated by adding all of the food group values and then classified as inadequate (<5 food groups per day) or adequate (≥5 food groups per day) [31].

#### Complete blood count and Kato-Katz examination.

Venous blood sample was withdrawn by experienced phlebotomists. Hemoglobin (Hb) was determined using an automated hematology analyzer for complete blood count (CBC). Fresh stool samples were collected and assessed for the presence of intestinal helminths using the microscopic Kato Katz method [32]. The results were classified as helminth infection if they contained ova for roundworm (*Ascaris lumbricoides*), whipworm (*Trichuris trichiura*), tapeworm (*Taenia saginata, Hymenolepis nana*), or blood flukes (*Schistosoma mansoni*). Blood and stool samples were kept in the cold chain (below 5°C) and delivered within 4 hours after collection to the main laboratory at the Faculty of Medicine, Universitas Mataram and UNRAM Hospital.

#### Anthropometry measurement.

Anthropometry standardization procedures were done to ensure enumerators met the required precision and accuracy. Mid-Upper Arm-Circumference (MUAC) was measured by using ergonomic circumference measuring tape (SECA 201) to determine the nutritional status of pregnant women. MUAC was measured on the upper arm, halfway between the olecranon acromion and olecranon processes by using flexible and non-stretchable tape to the nearest 0.1 cm.

### Statistical analysis

The overall statistical data analysis in this study is performed using SPSS version 20.0 software. Pregnant women’s characteristics were summarized using frequencies and percentages and presented using descriptive analysis. To ascertain if the continuous variables were normally distributed, the Kolmogorov-Smirnov test was performed. The frequency distribution of pregnant women stratified by anemia status was assessed using Chi-square test. Anemia in pregnancy (Hb < 11 g/dL) is the outcome of this study. The predictors were CED (MUAC < 23.5 cm), maternal age, household wealth index, family size, appetite status, dietary diversity, use of contraceptive methods, gravidity, and changes in household income and food expenditure. Logistic regression analysis was used to determine the odds ratio (OR) of each significant risk factor of anemia. If any variables have a p-value of 0.25 in bivariate analysis, they will be included in the binary logistic regression model (multivariable). All statistics were performed using 2-sided tests, statistical significance was considered at a p-value of less than 0.05, and OR with 95% confidence intervals (CI) were presented.

### Ethical considerations

The study was approved by the Research Ethical Review Committee of the Faculty of Medicine, Universitas Indonesia (Reference Number: KET-887/UN2.F1/ETIK/PPM.00.02/2019). All individuals provided written informed consent before participating in the study (or a witnessed thumbprint for those who were unable to provide a signature). Prior to the interview, the enumerators fully explained the study’s objectives and those who agreed to participate signed a written informed consent form. The participants were also informed that any data obtained from them would be kept anonymous and confidential through the use of codes (instead of any personal identifiers) and would be used only for purpose of the study. The study participants can withdraw their participation at any time of the study period without penalty.

## Results

Anemia was found in 40.8% of pregnant women in the third trimester. One third of study participants were young mothers aged <25 years and 1 in 2 mothers had a low education level of primary to middle school. Most mothers were housewives with 3–5 members living in the same household and majority of them were passive smokers. Approximately 10.5% of pregnant women had chronic energy deficiency (CED, indicated by MUAC <23.5 cm) and 6.5% were infected with intestinal helminth, most of which was *Trichuris trichiura* (see Table 1).

**Table 1:** Socio-economic characteristics and nutritional status of pregnant women in East Lombok district (N = 446).

Variables		n	(%)
Age of pregnant women (years)^a^		28.0 (10.0)	
	< 25	134	30.0
	25–29	121	27.1
	30–34	123	27.6
	≥ 35	68	15.2
Level of education	Primary to middle school	222	49.8
	Secondary school	178	39.9
	College and above	46	10.3
Employment status	Unemployed	328	73.5
	Employed	118	26.5
Occupation	Housewife/not working/ student	328	73.5
	Merchant or traders	42	9.4
	Agricultural-related work	31	7.0
	Professional work	23	5.2
	Private work	22	4.9
Household wealth index (tercile)^c^	Poor	150	33.6
	Middle	150	33.6
	Rich	146	32.7
Family size (person)	1–2	120	26.9
	3–5	304	68.2
	6 and above	22	4.9
Smoking status	No	84	18.8
	Yes	362	81.2
Hemoglobin concentration (g/dl)^a^		11.2 ± 1.3	
Anemia status	Anemic	182	40.8
	Non-Anemic	264	59.2
Mid Upper Arm Circumference or MUAC (cm)^a^		27.4 ± 13.0	
	≥ 23.5 (Normal)	399	89.5
	< 23.5 (CED)	47	10.5
Intestinal helminth infection	Absent (No)	417	93.5
	Present (Yes)	29	6.5
Types of helminth infection	*Trichuris trichiura*	27	6.1
	*Taenia saginata*	2	0.4
	*Hymenolepis nana*	2	0.4
	*Ascaris lumbricoides*	1	0.2
	*Schistosoma mansoni*	1	0.2
Food security status	Food secure (0–2)	313	70.2
	Food insecure (≥ 3)	133	29.8
Food taboos and/or aversions	No	146	32.7
	Yes	300	67.3
Appetite status	Decreased	67	15.0
	No change	105	23.5
	Increased	274	61.4
Dietary Diversity Score^b^		6.0 (4–7)	
MDD-W	Adequate (≥ 5)	320	71.7
	Inadequate (< 5)	126	28.3
Use of contraceptives	Never/ Not used (N)	173	38.8
	Ever Used (Y)	273	61.2
Planned and desired pregnancy	No	69	15.5
	Yes	377	84.5
Gravidity	Nulligravida (0)	113	25.3
	Primigravida (1)	152	34.1
	Multigravida (≥ 2)	181	40.6
Number of ANC Visits	<6	85	19.1
	≥ 6	361	80.9
ANC Visits (according to WHO guideline)	< 8 (Inadequate)	369	82.7
	≥ 8 (Adequate)	77	17.3
IFAS consumption (adherence)	Not consumed	124	27.8
	< 30 tablets/month	279	62.6
	≥ 30 tablets/month	43	9.6
Deworming medication	No	446	100
Animal rearing ownership	Poultry	173	38.8
	Livestock	36	8.1
	Fish pond	14	3.1
Government assistance	Yes, both cash and non-cash	75	16.8
	Yes, only cash	102	22.9
	Yes, only non-cash	54	12.1
	No	215	48.2
Changes in household income	No change or increased	113	25.3
	Decreased	333	74.7
Changes in household food expenditure	Decreased	129	28.9
	No change	135	30.3
	Increased	182	40.8
RCSI^b^		4.0 (1–9)	
	Low coping (0–4)	237	53.1
	Medium to high coping (≥ 5)	209	46.9

^a^Mean ± SD

^b^Median (25th-75th percentiles)

^c^Poor if the wealth index score ranges from –174.470 to –0.281; middle if the score range from –0.281 to –0.039; rich if the score range from –0.040 to 0.430

CED: Chronic energy deficiency; MDD-W: Minimum dietary diversity for women; ANC: Antenatal care; IFAS: Iron-folic acid supplementation; RCSI: Reduced coping strategy index

The proportion of food insecurity, food taboos, and inadequate dietary diversity were 29.8%, 67.3%, and 28.3%, respectively. Plant source foods (PSFs) and animal source foods (ASFs) were acquired mostly through cash purchases or as gifts from friends or relatives. More than half of the participants reported an increase in appetite status during the third trimester of pregnancy compared to the first trimester. About 39% of study participants had never or had not used any contraceptive method prior to their current pregnancy, 15.5% had unplanned pregnancies (self-perceived), and 40.6% were multigravida. Approximately 19.1% of women had insufficient ANC visits, with a total of fewer than 6 visits throughout pregnancy. Most of them did not adhere to the IFAS consumption guidelines, with less than 30 tablets consumed per month (62.6%). None of the women reported consuming deworming medication during their current pregnancy (see Table 1).

More than half of pregnant women received government assistance programs during the COVID-19 pandemic, in the form of cash and/or non-cash aid. Three-quarters of participants reported a decrease in household income, while 28.9% reported a decrease in household food expenditure. Nearly half of them reported medium to high coping strategies, indicating food insecurity (Table 1). The three most common strategies used by pregnant women were relying on less preferred and less expensive foods (65.9%), limiting adult intake to allow young children to eat (30.5%), and borrowing food or relying on help from friends or relatives (29.8%).

Our findings demonstrated that older women above 35 years (COR = 2.73; 95% CI: 1.50, 4.98), with low levels of education (COR = 2.64; 95% CI: 1.34, 5.22), multiparous (COR = 2.82; 95% CI: 1.73, 4.63), experienced decrease in household income during pandemic (COR = 2.31; 95% CI: 1.47, 3.63), and food insecurity (COR = 1.52; 95% CI: 1.01, 2.28) were more likely to employ medium to high coping strategies (RCSI ≥5). Furthermore, pregnant women who did not obtain any government assistance (COR = 0.53; 95% CI: 0.31, 0.90) were less likely to have higher RCSI scores. However, women who had ever used contraceptives prior to their current pregnancy (COR = 1.86; 95% CI: 1.26, 2.74) were associated with higher RCSI score.

In the bivariate analysis, the factors significantly associated with medium to high food coping strategies during the COVID-19 pandemic were the older age of mothers (aged ≥35 years, COR = 2.73; 95% CI: 1.50, 4.98), attained primary to middle school (COR = 2.64; 95% CI: 1.34, 5.22), ever-used contraceptives (COR = 1.86; 95% CI: 1.26, 2.74), were multiparous (COR = 2.82; 95% CI: 1.73, 4.63), food insecure (COR = 1.52; 95% CI: 1.01, 2.28), and experienced a decrease in household income during the pandemic (COR = 2.31; 95% CI: 1.47, 3.63). Otherwise, pregnant women who did not receive government assistance are less likely to employ medium-to-high coping strategies (COR = 0.53; 95% CI: 0.31, 0.90) compared with their counterparts.

The factors associated with anemia in pregnant women were determined using a logistic regression model (Table 2). In bivariate analyses, pregnant women under the age of 25 year were 1.8 times more likely than other age groups to develop anemia (COR = 1.83; 95% CI: 1.00, 3.32). Pregnant women with 3–5 family members were less likely to have anemia than women with 1–2 family members and more than 6 family members (COR = 0.57; 95% CI: 0.37, 0.87). Additionally, women who were nulligravida were more likely to have anemia (COR = 1.90; 95% CI: 1.18, 3.07) than multigravida or primigravida.

**Table 2:** Factors associated with anemia among pregnant women in East Lombok district (N = 446).

Variables		Anemia Status		COR (95% CI)	AOR (95% CI)^a^
		Yes	No		
		n (%)	n (%)		
MUAC	≥ 23.5 cm (Normal)	145 (38.0)	237 (62.0)	*1*	*1*
	< 23.5 cm (CED)	37 (57.8)	27 (42.2)	2.24 (1.31, 3.83)*	1.92 (1.06, 3.48)*
Intestinal helminth infection	Absent (No)	168 (40.3)	249 (59.7)	*1*	
	Present (Yes)	14 (48.3)	15 (51.7)	1.38 (0.65, 2.94)	**−**
Age of pregnant women	< 25	69 (51.5)	65 (48.5)	1.83 (1.00, 3.32)*	1.27 (0.54, 2.95)
	25–29	48 (39.7)	73 (60.3)	1.13 (0.61, 2.09)	1.01 (0.50, 2.02)
	30–34	40 (32.5)	83 (67.5)	0.83 (0.45, 1.54)	0.79 (0.41, 1.52)
	≥ 35	25 (36.8)	43 (63.2)	*1*	*1*
Level of education	Primary to middle school	82 (36.9)	140 (63.1)	0.70 (0.37, 1.32)	**−**
	Secondary school	79 (44.4)	99 (55.6)	0.95 (0.50, 1.82)	**−**
	College and above	21 (45.7)	25 (54.3)	*1*	**−**
Employment status	Unemployed	137 (41.8)	191 (58.2)	1.16 (0.76, 1.79)	**−**
	Employed	45 (38.1)	73 (61.9)	*1*	**−**
Household wealth index	Poor	69 (46.0)	81 (54.0)	1.54 (0.97, 2.46)	1.23 (0.74, 2.04)
	Middle	61 (40.7)	89 (59.3)	1.24 (0.77, 1.98)	1.08 (0.65, 1.79)
	Rich	52 (35.6)	94 (64.4)	*1*	*1*
Family size	1–2	60 (50.0)	60 (50.0)	*1*	*1*
	3–5	110 (36.2)	194 (63.8)	0.57 (0.37, 0.87)*	0.96 (0.54, 1.71)
	6 and above	12 (54.5)	10 (45.5)	1.20 (0.48, 2.99)	1.89 (0.70, 5.12)
Smoking status	No	38 (45.2)	46 (54.8)	*1*	**−**
	Yes	144 (39.8)	218 (60.2)	0.80 (0.50, 1.29)	**−**
Food security status	Food secure	132 (42.2)	181 (57.8)	*1*	**−**
	Food insecure	50 (37.6)	83 (62.4)	0.83 (0.55, 1.25)	**−**
Food taboos and aversions	No	58 (39.7)	88 (60.3)	*1*	**−**
	Yes	124 (41.3)	176 (58.7)	1.07 (0.71, 1.60)	**−**
Appetite status	Decreased	24 (35.8)	43 (64.2)	*1*	*1*
	No change	36 (34.3)	69 (65.7)	0.94 (0.49, 1.78)	0.83 (0.42, 1.62)
	Increased	122 (44.5)	152 (55.5)	1.44 (0.83, 2.50)	1.21 (0.68, 2.17)
MDD-W	Adequate (≥ 5)	119 (37.2)	201 (62.8)	*1*	*1*
	Inadequate (< 5)	63 (50.0)	63 (50.0)	1.69 (1.11, 2.56)*	1.58 (1.02, 2.45)*
Use of contraceptives	Never/ Not used (N)	86 (49.7)	87 (50.3)	1.82 (1.23, 2.69)*	1.58 (1.02, 2.45)*
	Ever used (Y)	96 (35.2)	177 (64.8)	*1*	*1*
Planned and desired pregnancy	No	30 (43.5)	39 (56.5)	1.14 (0.68, 1.91)	**−**
	Yes	152 (40.3)	225 (59.7)	*1*	**−**
Gravidity	Nulligravida (0)	59 (52.2)	54 (47.8)	1.90 (1.18, 3.07)*	0.92 (0.42, 2.02)
	Primigravida (1)	57 (37.5)	95 (62.5)	1.05 (0.67, 1.63)	0.83 (0.49, 1.41)
	Multigravida (≥ 2)	66 (36.5)	115 (36.5)	*1*	*1*
Number of ANC visits	< 6	35 (41.2)	50 (58.8)	1.02 (0.63, 1.65)	**−**
	≥ 6	147 (40.7)	214 (59.3)	*1*	**−**
IFAS consumption (adherence)	Not consumed	52 (41.9)	72 (58.1)	1.11 (0.54, 2.24)	**−**
	< 30 tablets/month	113 (40.5)	166 (59.5)	1.04 (0.54, 2.01)	**−**
	≥ 30 tablets/month	17 (39.5)	26 (60.5)	*1*	**−**
Government assistance	Yes, both cash and non-cash	26 (34.7)	49 (65.3)	*1*	*1*
	Yes, only cash	41 (40.2)	61 (59.8)	1.27 (0.68, 2.35)	1.23 (0.64, 2.37)
	Yes, only non-cash	18 (33.3)	36 (66.7)	0.94 (0.45, 1.97)	0.74 (0.34, 1.64)
	No	97 (45.1)	118 (54.9)	1.55 (0.90, 2.68)	1.10 (0.59, 2.05)
Changes in household income	No change or increased	51 (45.1)	62 (54.9)	*1*	**−**
	Decreased	131 (39.3)	202 (60.7)	0.79 (0.51, 1.21)	**−**
Changes in household food expenditure	Decreased	46 (35.7)	83 (64.3)	*1*	*1*
	No Change	60 (44.4)	75 (55.6)	1.44 (0.88, 2.37)	1.44 (0.85, 2.45)
	Increased	76 (41.8)	106 (58.2)	1.29 (0.81, 2.06)	1.53 (0.93, 2.51)
RCSI	Low coping (0–4)	99 (41.8)	138 (58.2)	*1*	**−**
	Medium to high coping (≥ 5)	83 (39.7)	126 (60.3)	0.92 (0.63, 1.34)	**−**

*1* set as a reference,

*Significant at p-value <0.05.

Only variables with p < 0.25 were retained in the final multivariable analysis model

^a^Adjusted for MUAC, age, household wealth index, family size, appetite status, MDD-W, use of contraceptives, gravidity, government assistance, and changes in household food expenditure.

COR: Crude odds ratio; AOR: Adjusted odds ratio; CI: Confidence interval

Chronic energy deficiency, non-use of contraceptive method, and inadequate dietary diversity were significant predictors of anemia (p < 0.05) in pregnant women after controlling for other confounders in multivariable analysis. Pregnant women with CED, as indicated by a MUAC of less than 23.5 cm, were 1.9 times more likely to have anemia in the third trimester (AOR = 1.92; 95% CI: 1.06, 3.48). Women who had never or had not used contraceptives prior to their current pregnancy were nearly 1.6 times as likely to have anemia (AOR = 1.58; 95% CI: 1.02, 2.45). Furthermore, inadequate dietary diversity, increased the risk of anemia in pregnant women by 1.6 times (AOR = 1.58; 95% CI: 1.02, 2.45) (see Table 2).

## Discussion

Maternal anemia remains a major problem in rural area of East Lombok, Indonesia. During this COVID-19 pandemic, decrease in household income and food expenditure were observed, along with medium-to-high coping strategies in half of the households. Chronic energy deficiency, inadequate dietary diversity, and non-use of contraception were significant determinants of anemia among pregnant mothers in the area.

The current study showed that the prevalence of maternal anemia was 40.8%, and is classified as severe public health issue by the WHO [7]. The proportion of anemia demonstrated by this study is lower than the national prevalence (48.9%) [5], yet higher than the global average of 36.5% [33]. While anemia prevalence remains high, this figure suggests reduction over the past two decades since a similar study conducted in 2001 amongst pregnant mothers in the third trimester in Lombok which showed anemia prevalence of 60% [34].

The proportion of CED in our study was 10.5%, which was lower than the national average of 17.3% and the sub-national rate in East Lombok of 20.2%. In terms of the high proportion of women of reproductive age with CED, our study area, i.e., NTB Province ranks eighth out of 34 provinces in Indonesia, indicating that maternal malnutrition remains a major public health concern [35]. Our study found that pregnant women with CED were 1.8 times more likely to develop anemia. This is consistent with a meta-analysis of studies in Indonesia, in which chronic energy deficiency was found to be the strongest predictor of anemia during pregnancy [36]. Malnutrition during pregnancy may be caused by poor dietary intake [3], low pre-pregnancy BMI (<18.5 kg/m^2^) [37], accompanied by infections and infestations, which is common in LMICs [38].

The present study found that 28% of the pregnant women had inadequate dietary diversity (DD) which was associated with a higher odd of anemia. Dietary diversity has been used to represent dietary quality and the probability of micronutrient adequacy in a diet [39]. Our findings are in line with a recent study in India which revealed that women with a more diverse diet had a 30% lower risk of anemia (OR= 0.7, 95% CI = 0.5–0.98) [36]. Similarly in Bangladesh, dietary diversity can predict the adequacy of micronutrient intakes in pregnant adolescent girls and women [40]. In our study, inadequate dietary diversity is not associated with food security, i.e., among a quarter of food secure women in the current study were still reported to have inadequate dietary diversity. This indicated that availability of foods in the household did not translate into adequate dietary diversity. The gap between food availability and consumption suggest the importance of nutrition education that promote dietary diversity with locally available and accessible foods. In addition, since iron, folate, and calcium are the typical problem nutrients among pregnant mothers in Indonesia, promoting locally available foods rich in iron, folate, and calcium using food-based recommendation is potential to improve both dietary diversity and nutrient adequacy in these mothers.

In our study, women who utilized contraception prior to pregnancy have a lower risk of anemia, which is in line with previous study in Africa [41]. According to the 2017 Indonesia DHS, contraceptive use (all methods) has slightly increased from 62% in 2012 to 64% in 2017 [42]. Approximately 40% study participants had not used any kind of contraception prior to their current pregnancy, which could be explained by young and first-time mothers. In Indonesia, the trend for modern contraceptive use, among married women increased with age (most users are aged 35–39), multiparity (3–4 live births), and a quintile lower middle-class wealth [42]. Data from East Lombok statistics office showed that contraceptive users decrease with wealth, i.e., 78%, 76% and 65% in bottom 40, middle 40 and top 20 percentile, respectively [43].

The findings of this study align with those of Rana et al. (2016), who examined the impact of family planning on women’s anemia and child undernutrition using aggregate data from the World Bank, UNICEF, and the Economist Intelligence Unit. Their OLS regression results revealed a significant independent effect of contraceptive prevalence rate (CPR) on women’s anemia (β = −0.35, p < 0.01) [44]. Similarly, a cross-sectional study among pregnant women in Ethiopia found that anemia was 2.5 times more likely in unplanned pregnancies compared to planned ones (AOR = 2.5, 95% CI: 1.4, 4.42) [45]. Family planning enables women to postpone marriage, begin childbearing at an optimal age, maintain optimum inter-birth intervals, and limit the number of children they have. The use of family planning increases the likelihood of nutrient recovery (e.g., iron, folate) in the mother’s body, thereby supporting the nutritional demands of pregnancy and lactation [44]. Enhancing access to family planning, particularly through the promotion of contraceptive use, is essential as it enables women to improve their nutritional status, prevent nutrient depletion, and contribute to better pregnancy outcomes [44].

Among women who did not use any contraceptive methods, the majority (57.5%) are young (aged < 25 years), first-time mothers with little to no prior pregnancy experience. In bivariate analysis, young pregnant women (< 25 years) were found to have a higher risk of anemia (COR = 1.83; 95% CI = 1.00, 3.32; p = 0.049), suggesting that this issue should also be considered. Similarly, a study conducted at public health facilities in Southern Ethiopia found that pregnant women aged 20–24 were four times more likely to be anemic (95% CI: 1.08, 14.90) than other age groups [46]. Young women (aged 15–24) in particular have increased nutritional needs to support their physiological needs for rapid growth [47], and inadequate nutrition prior to conception will eventually increase the likelihood of nutritional deficiencies, including anemia [48].

More than one-quarter of the women in this study had never used or had not used any kind of contraceptive method prior to their current pregnancy. Moreover, our results revealed a modest association between non-use of contraceptives and anemia in pregnancy (AOR = 1.56; 95% CI: 1.01, 2.40; p = 0.047). According to a large study of 12,981 women of childbearing age in Ethiopia, current usage of modern contraception has lowered the odds of anemia by 50% when compared to non-users. Individual modern contraceptive use, including injectables (AOR = 0.59; 95% CI: 0.24 to 1.79), OCP (AOR = 0.6; 95% CI: 0.45, 1.12), and implant (AOR = 0.72; 95% CI: 0.60 to 0.86), was demonstrated to significantly reduce the risk of anemia [72]. Contraception use, especially in post-partum period, is critical for improving maternal and child survival since it helps to improve optimal birth spacing and prevent unplanned pregnancies, and has been linked to a 44% reduction in maternal mortality and a 21% reduction in under-five mortality in low-income countries [73].

Half (52%) of pregnant women in our study received government poverty alleviation programs which consist of conditional cash transfers and/or food aid. During the COVID-19 pandemic, government of Indonesia under *Program Keluarga Harapan* (PKH Program) provided conditional cash money for poor families with pregnant mothers and/or children 0–6 months [49]. Another program called *Bantuan Pangan Non Tunai* (BPNT Program) provided non-cash support for poor families in the form of rice, egg, poultry, meat and vegetables. These programs were also likely to have protected mothers from lower socioeconomics for better access to basic healthcare facilities, including antenatal care [50]. This is explained by more than three-quarters of women completed a minimum of six antenatal care visits throughout pregnancy, especially in the midst of the COVID-19 pandemic, as recommended by the Ministry of Health Indonesia (i.e., 2, 1 and 3 times in first, second and third trimesters, respectively) [51].

During the antenatal care visit, pregnant women are typically provided with iron folic acid supplementation (IFAS), as one of Indonesian MoH program to prevent anemia. The WHO recommends pregnant women to take at least 90 tablets of IFAS throughout pregnancy. Our study discovered that more than half of the respondents did not consume sufficient amounts of IFAS tablets during the past one month, which mean these women would not achieve the recommended iron-folic acid intake. Previous studies have explained that this issue of low compliance may be attributed to low socioeconomic and education level of pregnant women, limited access to the prenatal health care system, and inadequate counseling regarding the benefits of supplement use [52]. Similar to the family planning program, it should be considered to include compliance to IFAS as prerequisite in the government assistance program.

Most women in our study reported a decrease in household income, increase in food expenditure and half of them had moderate to high coping strategies as a result of the COVID-19 pandemic. Studies in developing countries discovered that individuals experienced significantly soaring food prices and decreased income as a result of the economic downturn during the pandemic [53,54]. Poor households that spend a large portion of their income on food and have few coping strategies are vulnerable to food insecurity, which will likely affect their dietary intake (for both quantity and quality) since their purchasing power has declined [54,55]. Therefore, ensuring the affordability of nutrient-dense foods, in addition to food availability, is critical.

Our hypothesis is that pregnant women with medium to high coping strategies which indicated food insecurity are more likely to be anemic, particularly during COVID-19 pandemic. The study findings, however, found no relationship between these variables. Our data showed that higher RCSI, while associated with lower maternal education and lower wealth index, was associated with higher contraceptive users which was protective against anemia based on the multivariable analysis. We discovered that the rural community in East Lombok is robust enough to overcome the condition of economic shock during the pandemic, since most (70.2%) were food secure and had adequate dietary diversity (71.7%). The provision of conditional cash transfers and food aid for poor households, which were received by more than half of study participants, was likely contribute to improve household food consumption and dietary diversity [56].

Our findings emphasize that nutrition-specific interventions are critical to tackling the anemia problem, given that inadequate dietary diversity and chronic energy deficiency were found to be direct determinants of anemia in this study. Our findings suggest to strengthened the current programs in two ways. Firstly, conditional cash transfers require poor households to comply with IFAS and family planning. Secondly, nutrition education for pregnant mothers through antenatal care visits and maternal classes, should promote food-based dietary recommendations and iron-folic acid supplementation.

This study is a community-based study which, to the best of our knowledge, is the first study in Indonesia to investigate the determinants of anemia in pregnant women during the COVID-19 pandemic. Most of the previous studies were institutional-based research that investigated pregnancy outcomes in women infected with SARS-CoV-2. Our study included solely pregnant women in the third trimester and *Sasaknese* ethnicity (as of the main observational cohort study), hence these results may not be generalizable to women in other trimesters of pregnancies and other regions. Previously, we reported about 70% of under two children have iron-lowering allele in TMPRSS6 [57] which contribute to anemia, however, we did not assess this genotype in the current study, which is limitation of this study. Finally, due to the cross-sectional nature of the study, the authors were unable to establish a causal relationship between the independent variables and anemia.

## Conclusions

The prevalence of anemia among pregnant women in East Lombok during COVID-19 pandemic remains high, and it is considered a major public health problem. Our study discovered that chronic energy deficiency, inadequate dietary diversity, and non-use of contraceptives were factors associated with anemia. Despite decreased income, increased food expenditure and increased RCSI, these were not found to be significant risk factors for anemia, potentially due to the existing cash and non-cash support for poor families. The findings from our study highlight the importance of improving anemia control program among pregnant women in rural areas like East Lombok as well as improving policy to strengthen their resilience to natural phenomenon such as COVID-19. Nutrition specific intervention including nutrition education which promote locally available and accessible nutrient-dense foods, and nutrition sensitive intervention with social safety nets such as conditional cash transfers and food aid, are expected to contribute to anemia prevention among pregnant women during the period of economic shock in this low-resource settings.

## Supporting information

S1 File Human participation research checklist. (DOCX)

## Abbreviations

ANC: antenatal care
ASF: animal source foods
CED: chronic energy deficiency
DMPA: depot-medroxyprogesterone acetate
IFAS: iron-folic acid supplementation
IYCF: infant and young child feeding
MUAC: mid-upper arm circumference
RCSI: reduced coping strategy index
PSF: plant source foods
UKRI-GCRF: United Kingdom Research and Innovation – Global Challenges Research Fund
WASH: water, sanitation and hygiene
WRA: women of reproductive age
